# Supplementary material for: Molecular and cellular immune features of aged patients with severe COVID-19 pneumonia
Source: Commun Biol. 2022 Jun 16;5:590. doi: 10.1038/s42003-022-03537-z (PMC9203559; doi:10.1038/s42003-022-03537-z)
Supplement: Supplementary file 2 — Supplementary Material [file 42003_2022_3537_MOESM2_ESM.pdf]

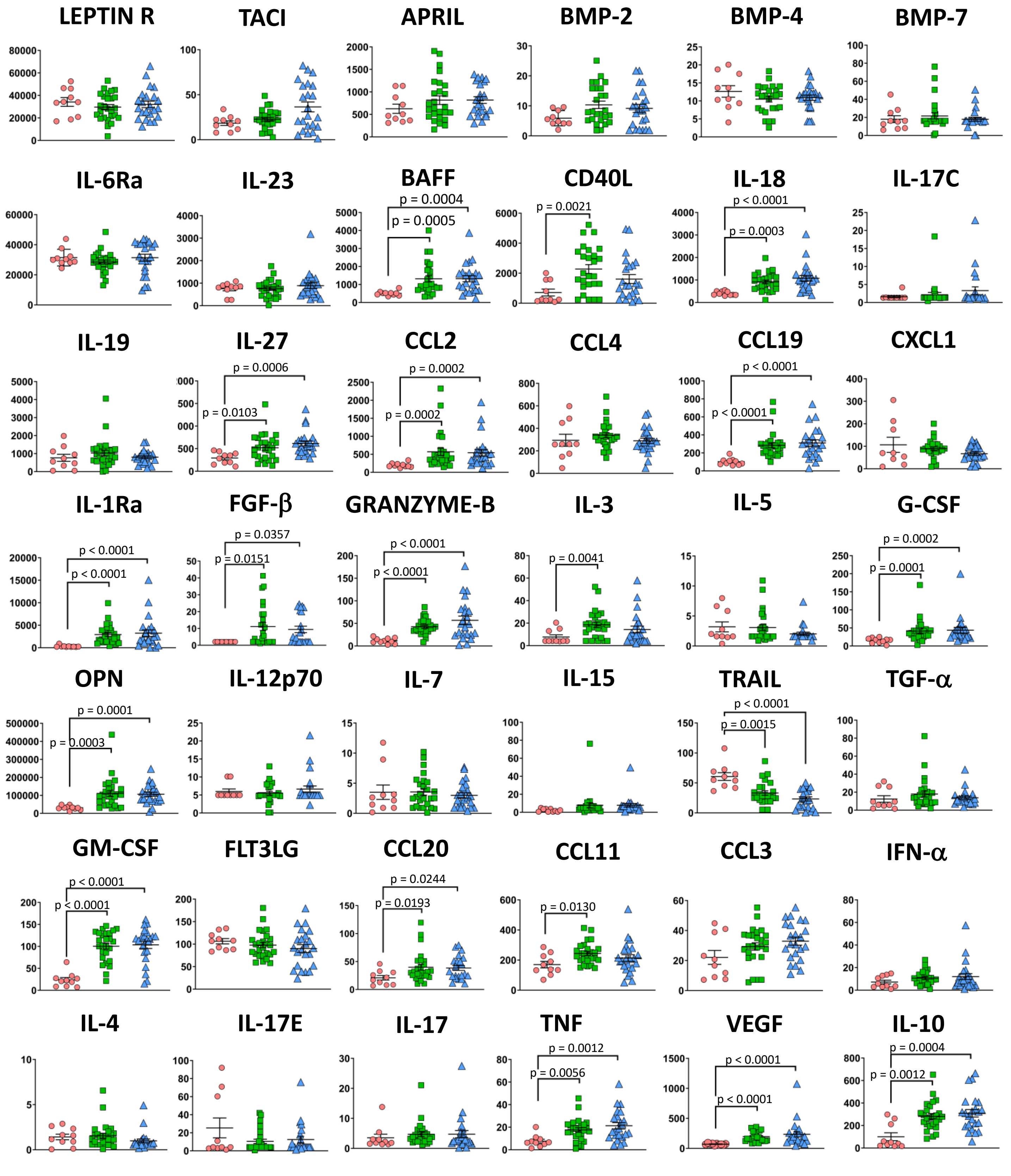

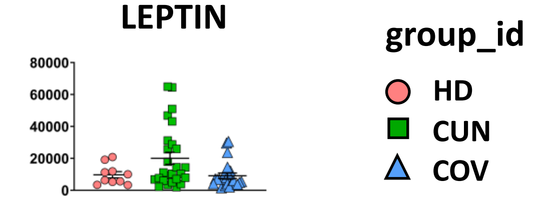

Supplementary Figure 1.
Dotplots showing 43 out of 62 measured plasmatic cytokines during severe SARS-CoV-2 infection among healthy donors (HD, n=10), COVID-under (CUN, n=26) and COVID-over (COV, n=23). The y-axis indicates pg/ml. Scatter plots show individual values, mean ± SEM. Statistical differences among values were assessed using Kruskal-Wallis test with Benjamini-Hochberg correction for multiple comparisons; only significant p-values are indicated.

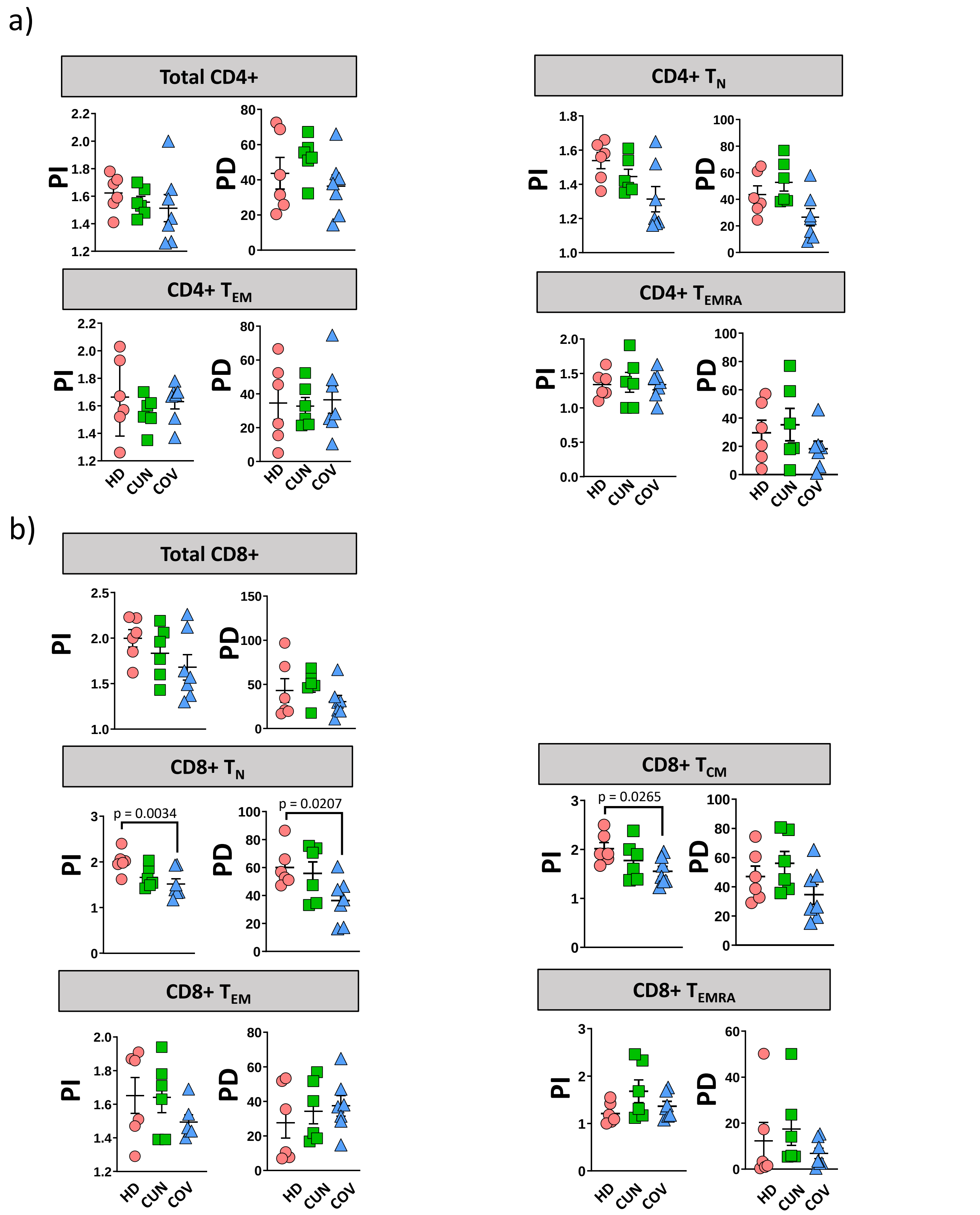

Supplementary Figure 2.

a) Dotplots show the results of CFSE dilution in CD4+ T cells of healthy donors (HD, n=6), COVID-under (CUN, n=6) and COVID-over (COV, n=7) patients after 16h of PBMC stimulation with 1 µg/mL of anti-CD3 plus anti-CD28. Naïve (TN), effector memory (TEM), effector memory re-expressing CD45RA (TEMRA); b) Dotplots show the results of CFSE dilution in CD8+ T cells of healthy donors (HD, n=6), COVID-under (CUN, n=6) and COVID-over (COV, n=7) after 16h of PBMC stimulation with 1 µg/mL of anti-CD3 plus anti-CD28. Naïve (TN), central memory (TCM), effector memory (TEM), effector memory re-expressing CD45RA (TEMRA). Paired dotplots show the proliferation index (PI) and percentage of divided cells (PD). Statistical differences among values were assessed using Kruskal-Wallis test with Benjamini-Hochberg correction for multiple comparisons; only significant p-values are indicated.

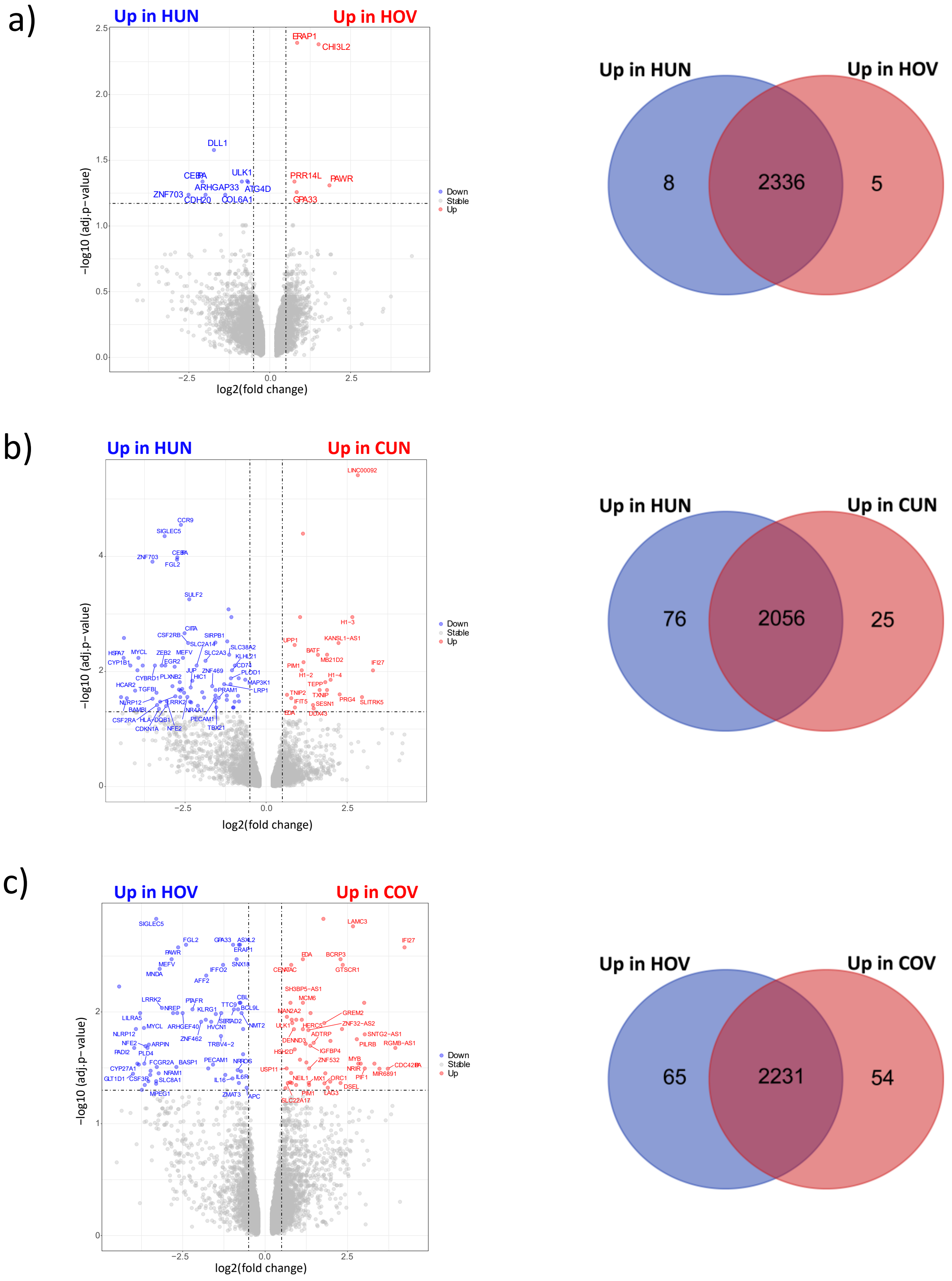

Supplementary Figure 3.

a) Volcano plot (left) shows the DEGs among CM CD4+ T cells from young healthy subjects (HUN) and aged healthy subjects (HOV). Venn diagram (right) shows the data summary of DEGs between HUN and HOV. Red indicates the upregulated genes in HOV, while blue the up-regulated genes in HUN; b) Volcano plot (left) shows the DEGs among CM CD4+ T cells from young healthy subjects (HUN) and COVID-under (CUN) samples. Venn diagram (right) shows the data summary of DEGs between HUN and CUN. Red indicates the upregulated genes in CUN, blue those upregulated in HUN; c) Volcano plot (left) shows the DEGs among CM CD4+ T cells from aged healthy subjects (HOV) and COVID-over (COV). Venn diagram (right) shows the data summary of DEGs between HOV and COV. Red indicates the upregulated genes in COV, in blue those upregulated genes in HOV. We report here as significant those genes that had FDR <0.05 and a log2FC > 0.5.

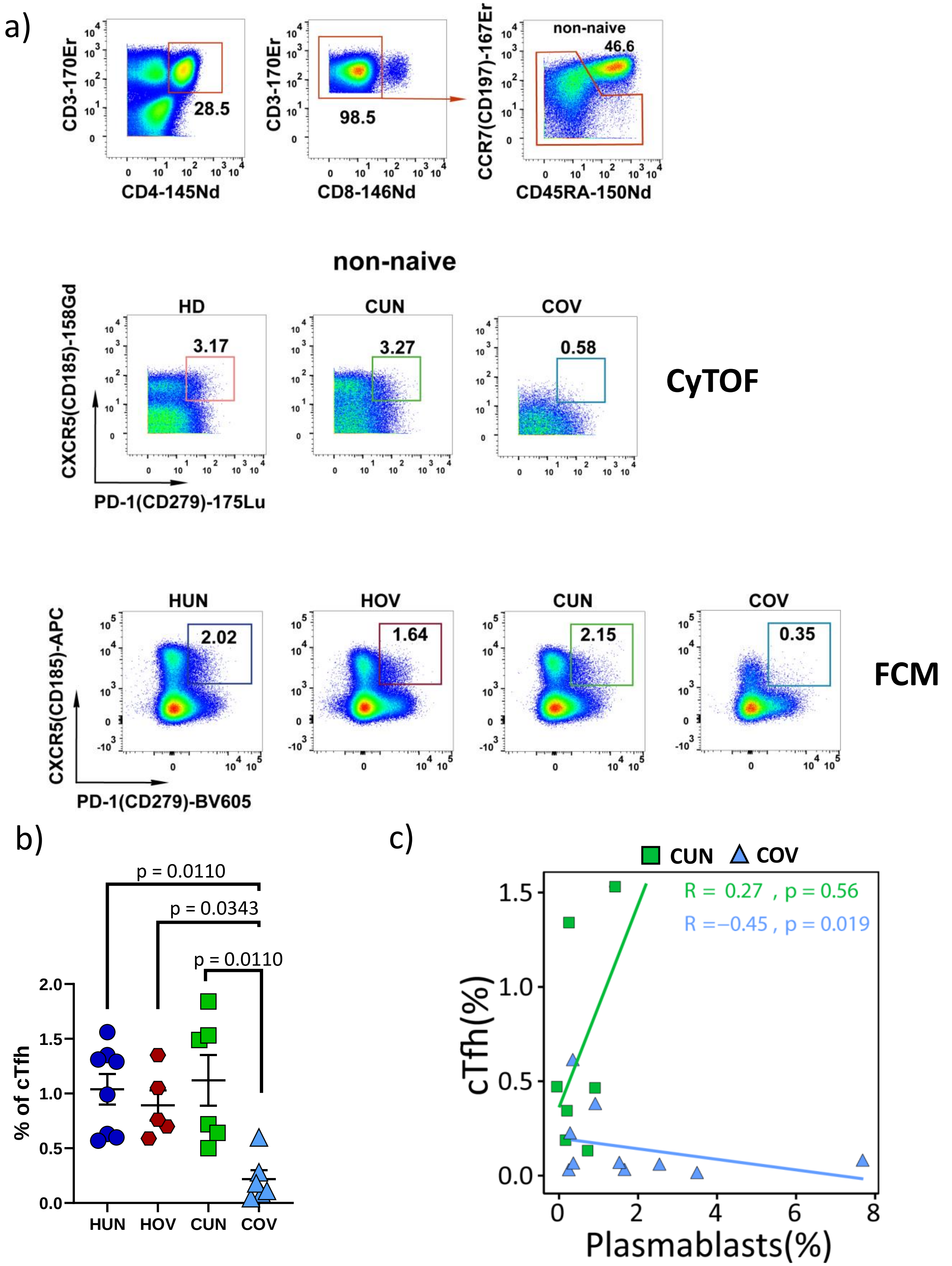

Supplementary Figure 4.

a) Representative gating strategy used to identify circulating CD4⁺ T follicular helper (cTfh) by CyTOF and flow cytometry (FMC). cTfh cells were identified by the coexpression of CXCR5 and PD-1 in non-naïve CD4⁺ T cells; b) Dot plot shows the percentage of cTfh identified using flow cytometry in young healthy donors (HUN, n=8), aged healthy donors (HOV, n=5), COVID-under (CUN, n=6) and COVID-over (COV, n=6). Statistical differences among values were assessed using Kruskal-Wallis test with Benjamini-Hochberg correction for multiple comparisons; only significant p-values are indicated. c) Spearman correlation between the percentage of cTfh and circulating plasmablasts in both CUN and COV patients. The percentage of cTfh cells and circulating plasmablasts used for the correlation are to those obtained from unsupervised analysis of PBMCs (as reported in Fig.2).

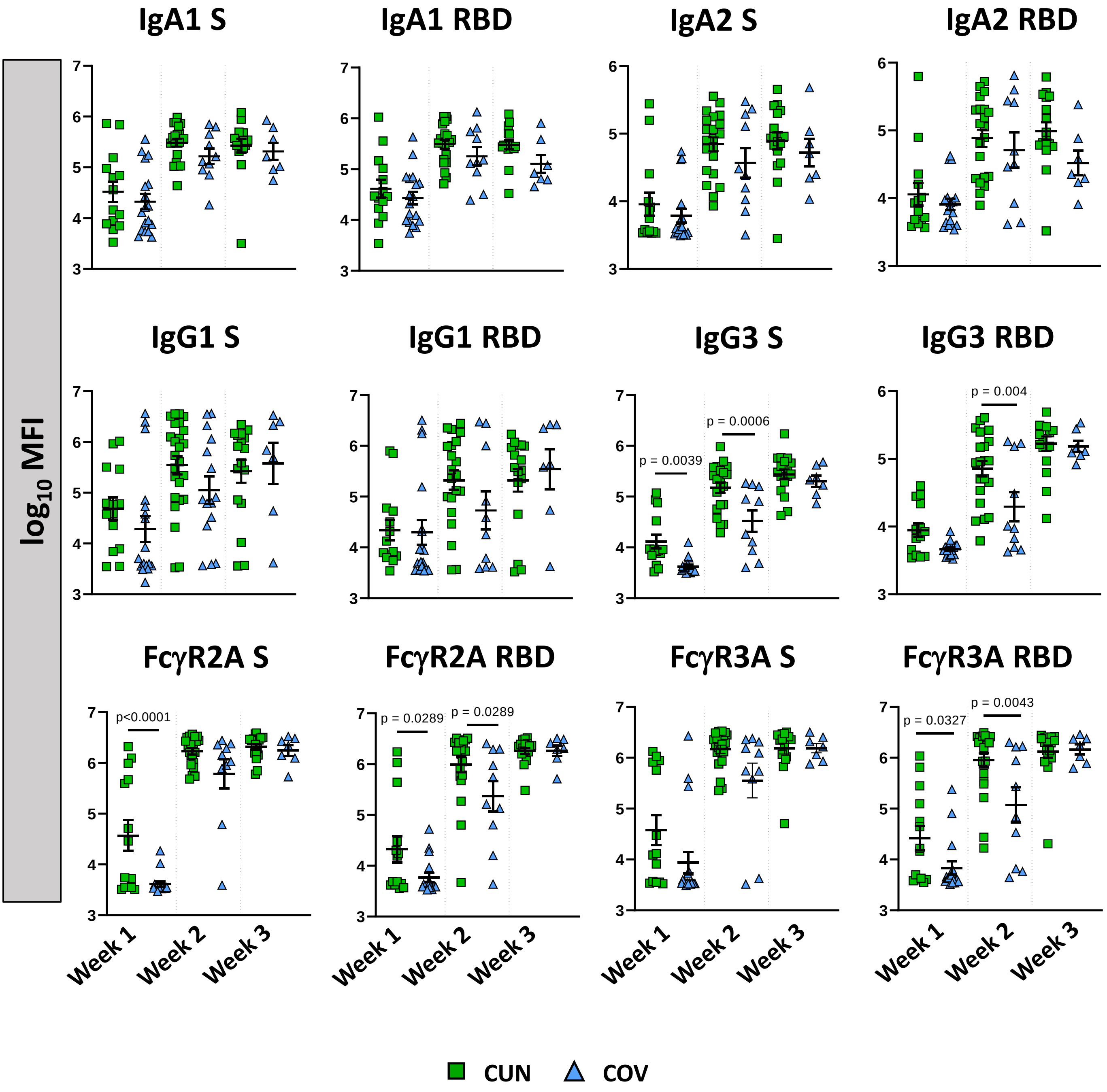

Supplementary Figure 5.
Immunoglobulin titers (IgA and IgG subtypes) and Fc receptors of severe COVID-under (CUN, green squares, n=21) and COVID-over (COV, azure triangles, n=18) over the course of 3–7, 12–14, and 15-21 days after infection. The solid black line represents the mean and SEM. A two-way ANOVA test was used to evaluate statistical differences across groups for all intervals followed by Benjamini-Hochberg correction. P-values are reported in the figure. The deceased patients across the two groups were n=2 for CUN and n=11 for COV.

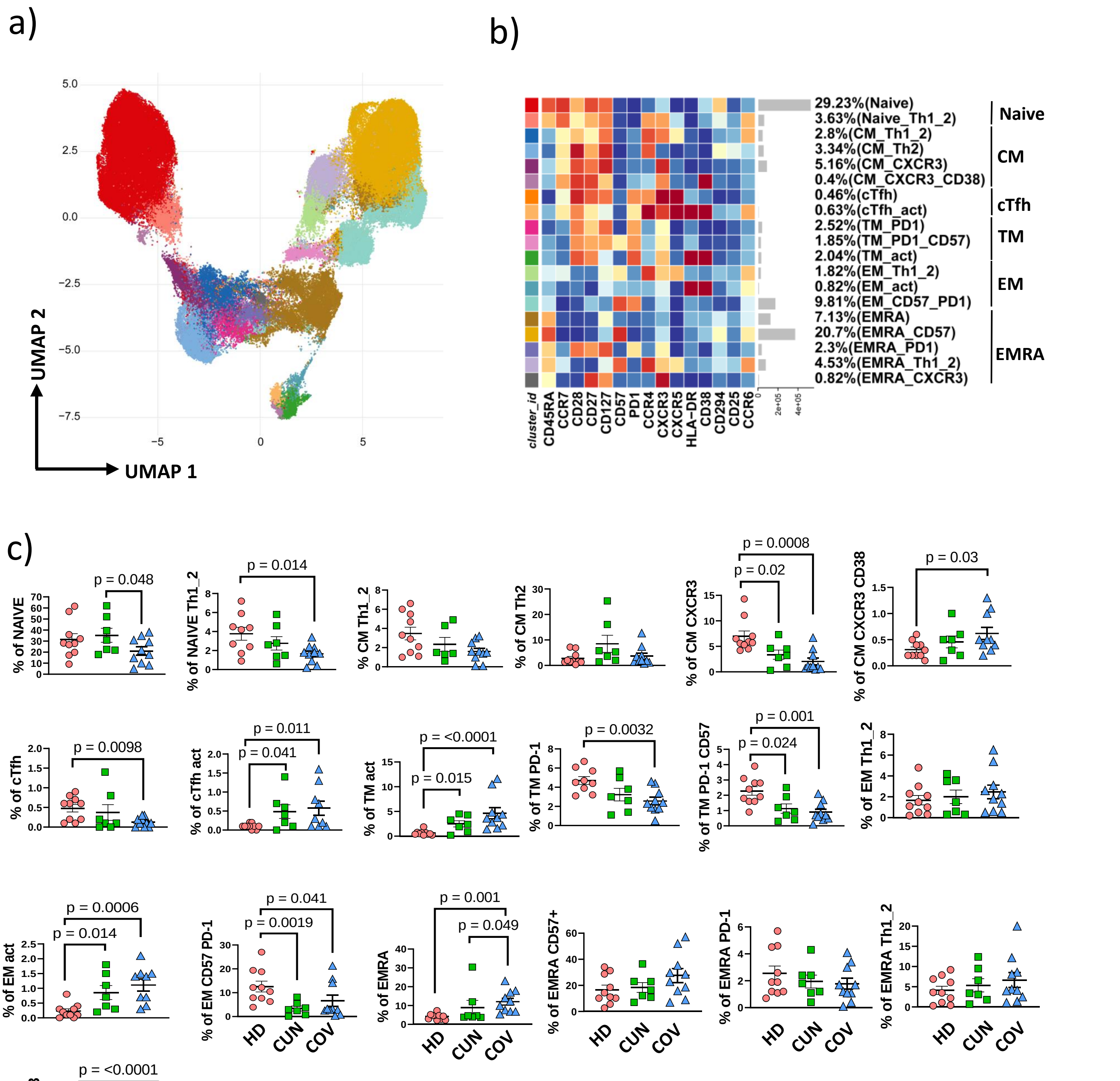

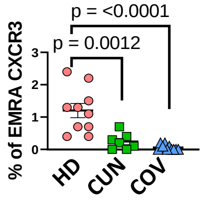

Supplementary Figure 6.
a) UMAP plot showing the 2D spatial distribution of 1,816,526 CD8+ T cells from healthy donors (HD, n=10), COVID-under (CUN, n=7) and COVID-over (COV, n=10) embedded with FlowSOM clusters; b) CD8+ T cells heatmap showing the median marker intensities of the 15 lineage markers across the 19 cell populations obtained with FlowSOM after the manual metaclusters merging. CM, central memory; TM, transitional memory; EM, effector memory; EMRA, effector memory re-expressing the CD45RA; cTfh, circulating T follicular helper cells. The black bar on the right is used to group subpopulations with similar immunophenotype; c) Dotplots show the relative cells percentage of the 19 clusters among healthy donors (HD; salmon circle; n = 10), severe young SARS-CoV-2 patients (CUN; green squares; n=7) and severe aged SARS-CoV-2 patients (COV; azure triangles; n=10). The central bar represents the mean ± SEM. GLMM test was used for the statistical analysis. Exact p-values are reported in the figure.

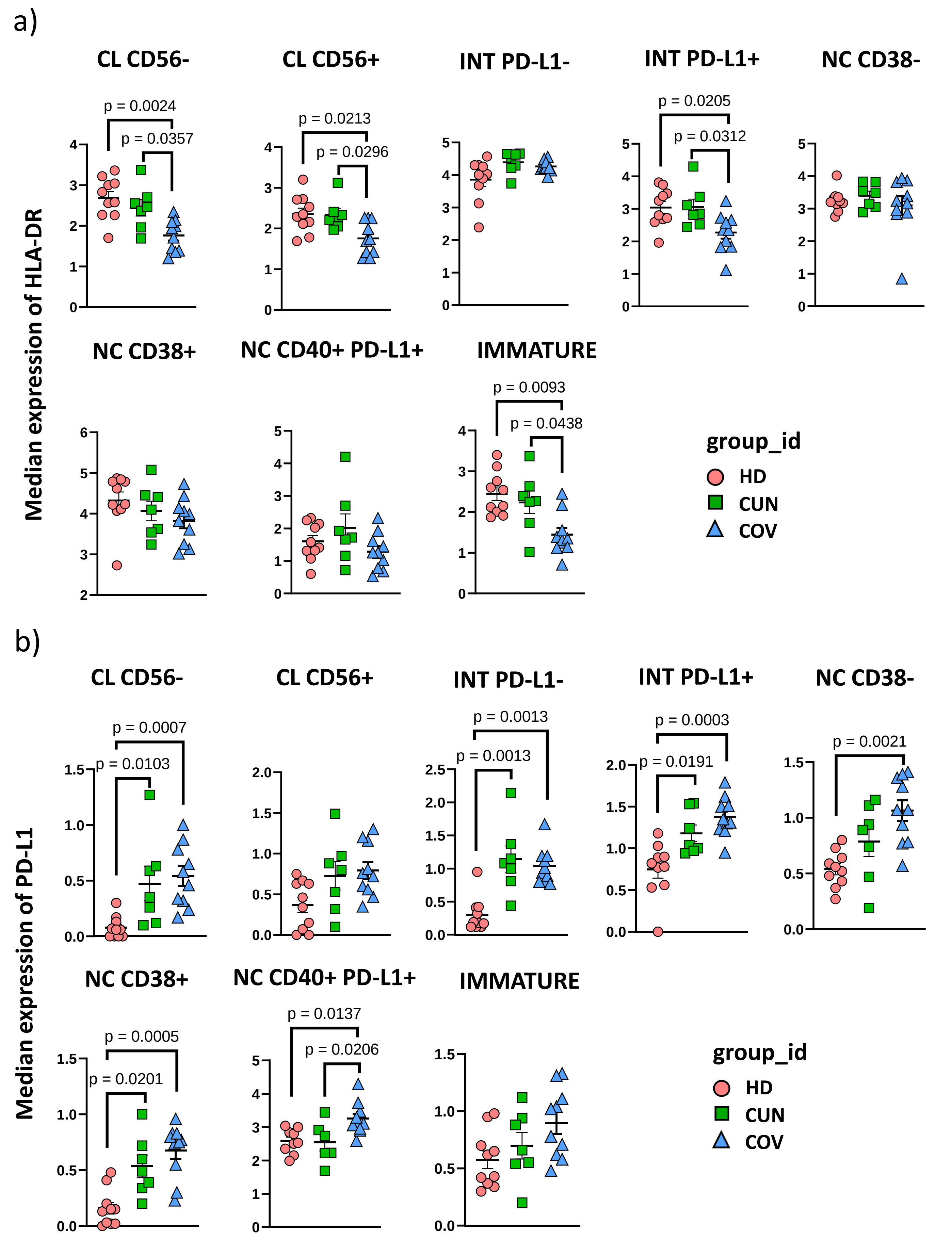

Supplementary Figure 7.

Dot plots showing the median expression (number of molecules) of HLA-DR (a) and PD-L1 (b) within monocyte clusters from healthy donors (HD, n=10), COVID-under (CUN, n=7) and COVID-over (COV, n=10). Statistical differences among values were assessed using Kruskal-Wallis test with Benjamini-Hochberg correction for multiple comparisons; only significant p-values are indicated. CL, classical monocytes; INT, intermediate monocytes; NC, non-classical monocytes.

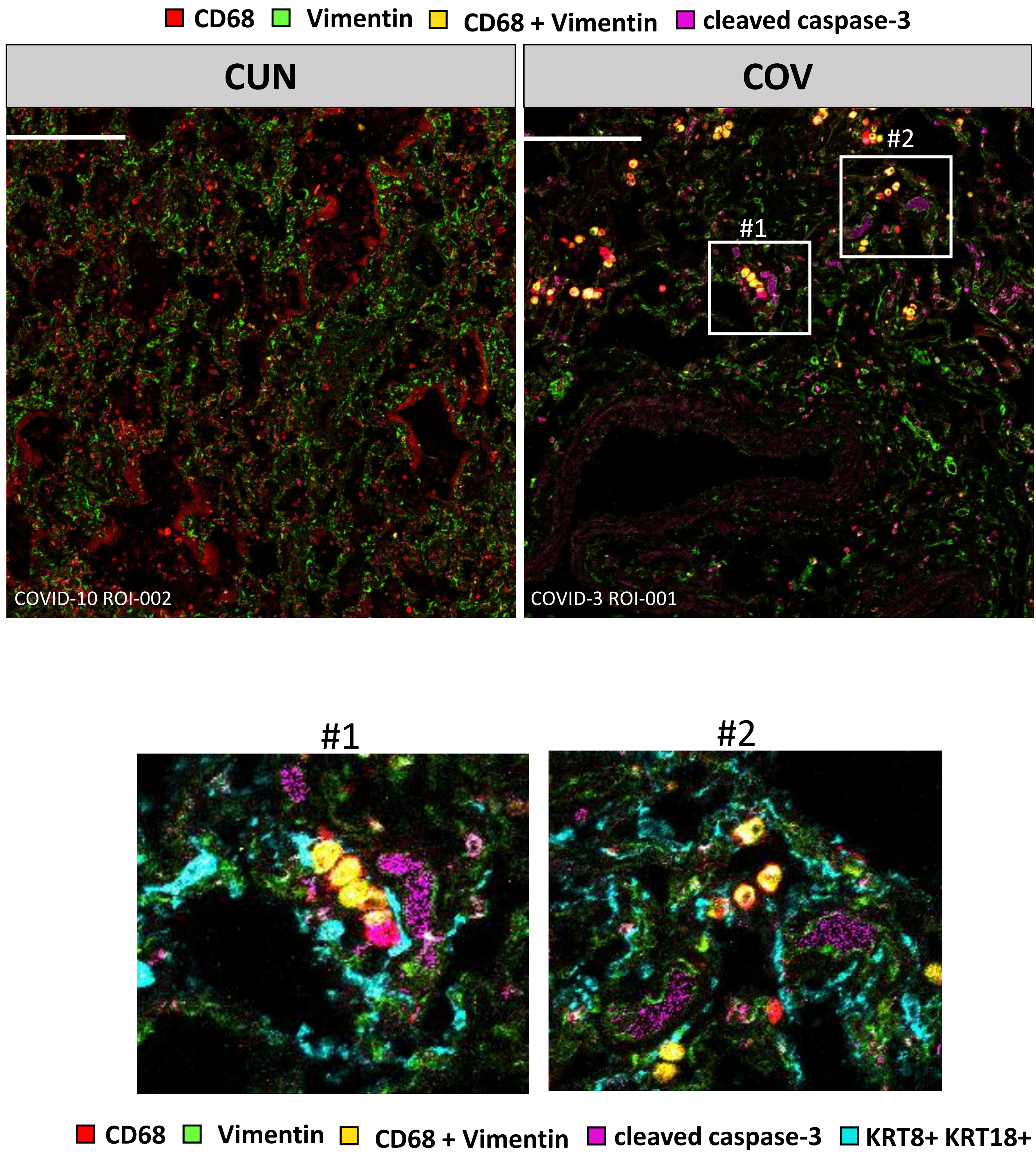

Supplementary Figure 8.
Spatial co-localization of activated macrophages expressing simultaneously CD68 (red dots), vimentin (yellow dots) and apoptotic sites (cleaved caspase-3, violet dots) in lung biopsies from COVID-under (CUN) and COVID-over (COV). Magnified images (#1 and #2) shows the colocalization of apoptotic sites with alveolar epithelial cells expressing keratin 8 and keratin 18 (KRT8+ KRT18+,cyan dots) in lung biopsies from COV. Scale bar 200 µm. Region of interest (ROI) and samples used are reported in the figure.

a)

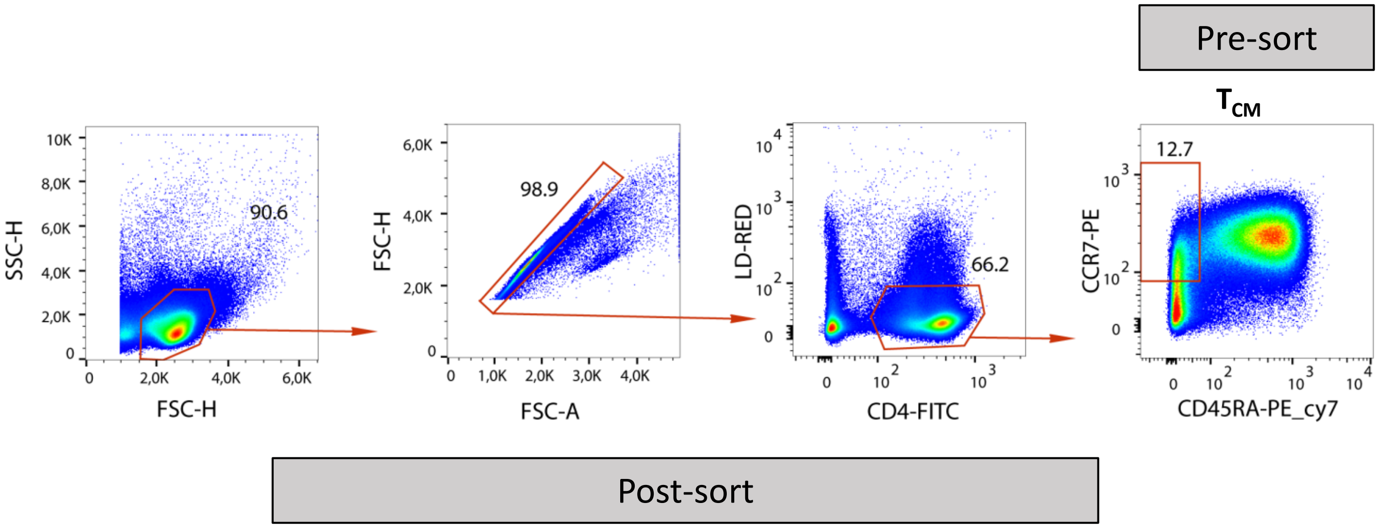

b)

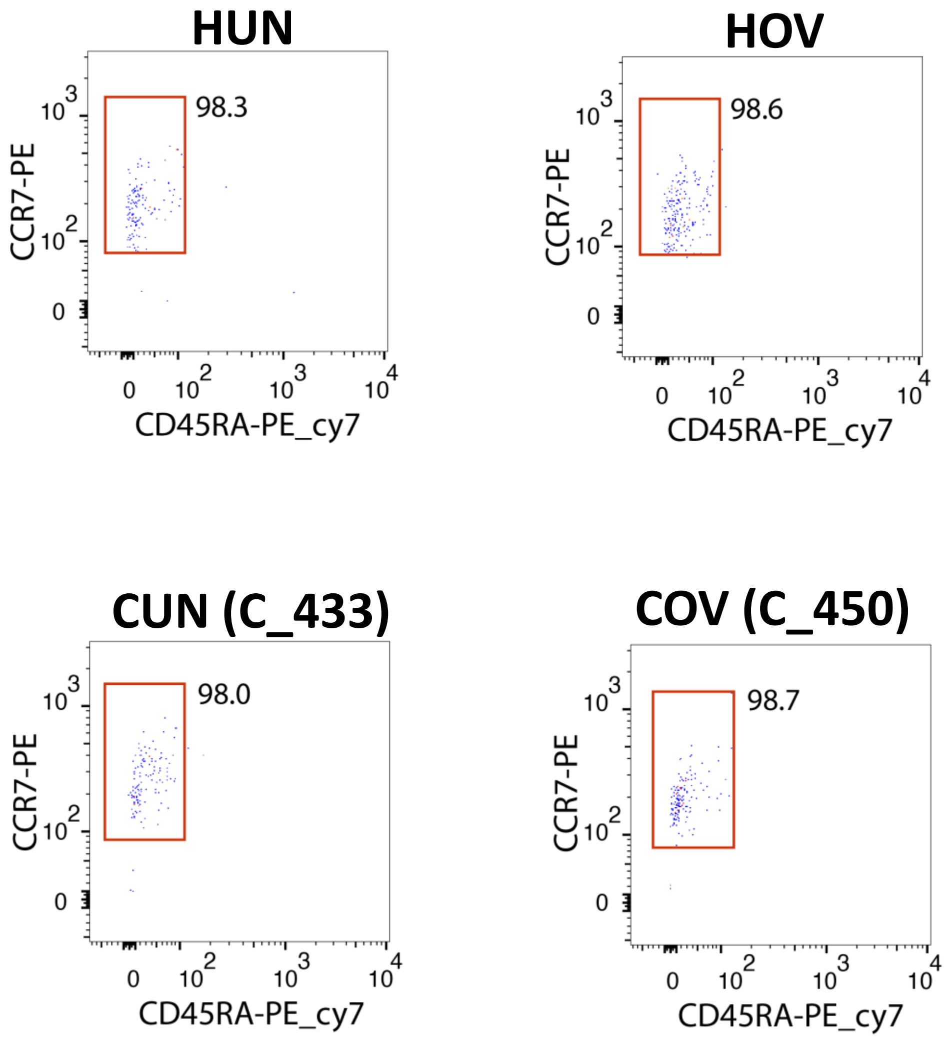

Supplementary Figure 9.

a) Gating strategy used to identify and sort CD4+ T_{CM} from purified $CD2^{+}$ cells; b) Representative dotplots depicting the purity of sorted CD4+ T_{CM} in young healthy subjects (HUN), aged healthy subjects (HOV), COVID-under (CUN) and COVID-over (COV).

a)

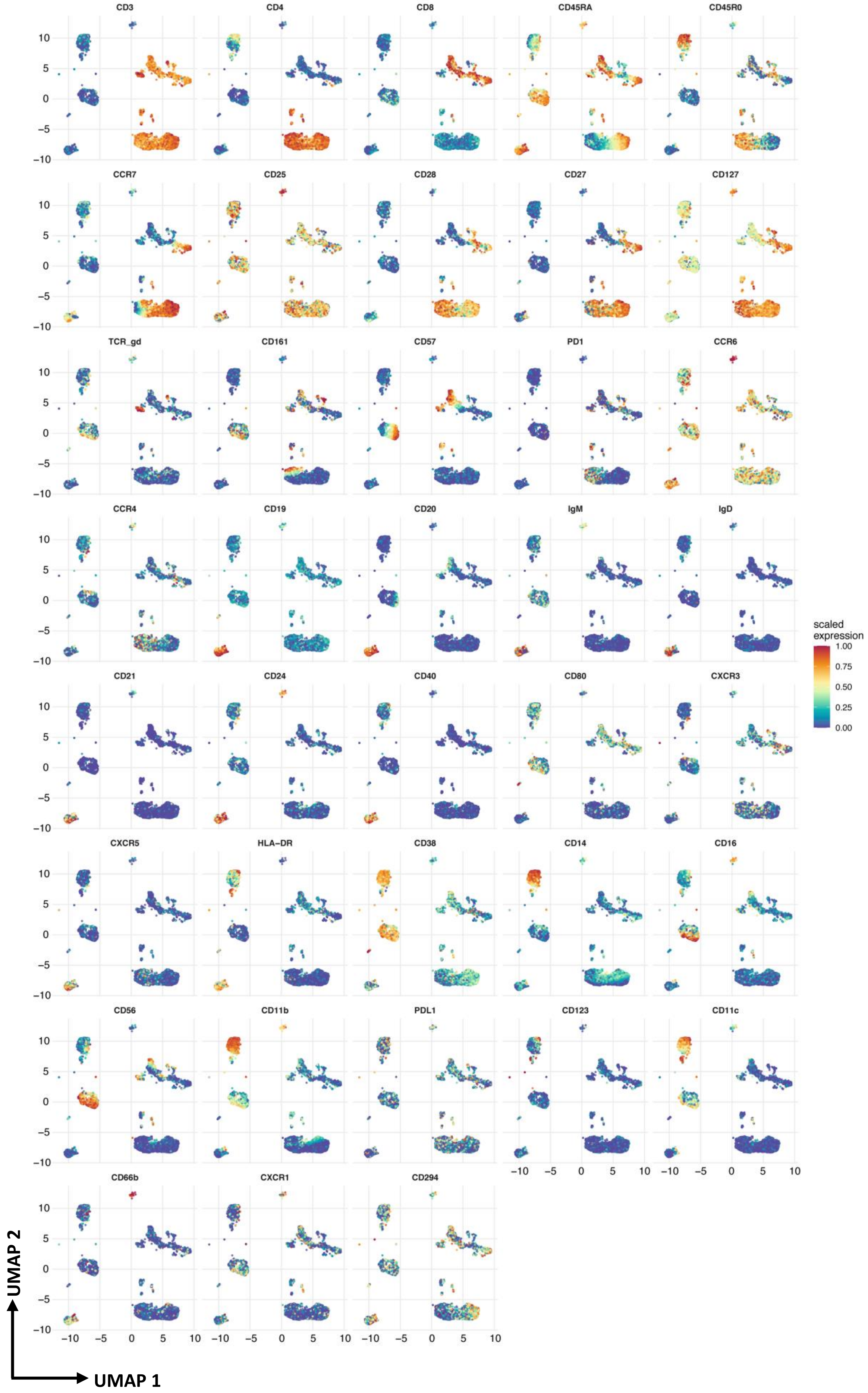

Supplementary Figure 10.
UMAP graphs colored by the expression of 38 markers used for PBMC phenotyping.

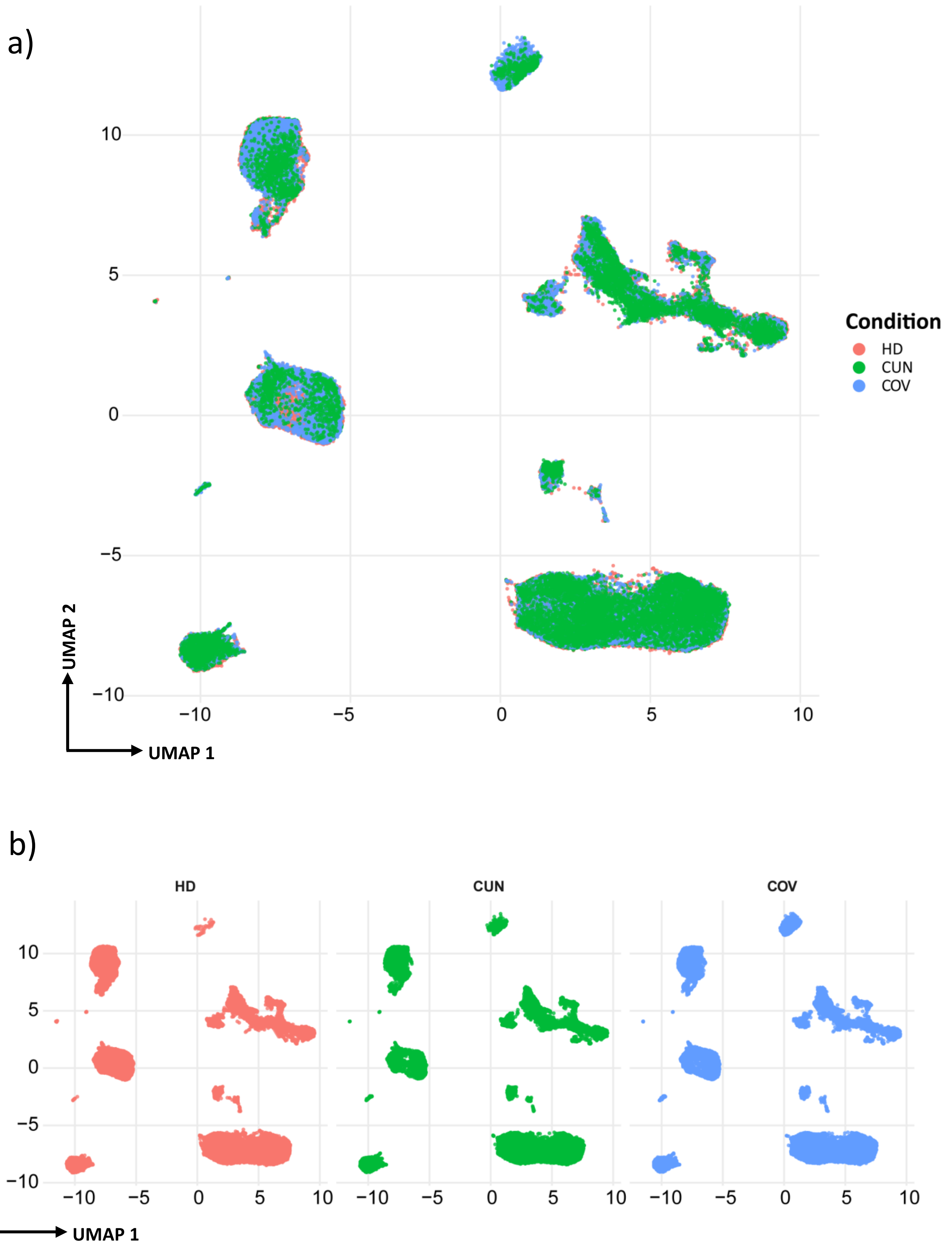

Supplementary Figure 11.
a) UMAP graph overlaid for multiple condition; b) Projection of UMAP graph stratified by condition. The condition levels are referred to: healthy donors (HD), COVID-under (CUN) and COVID-over (COV).

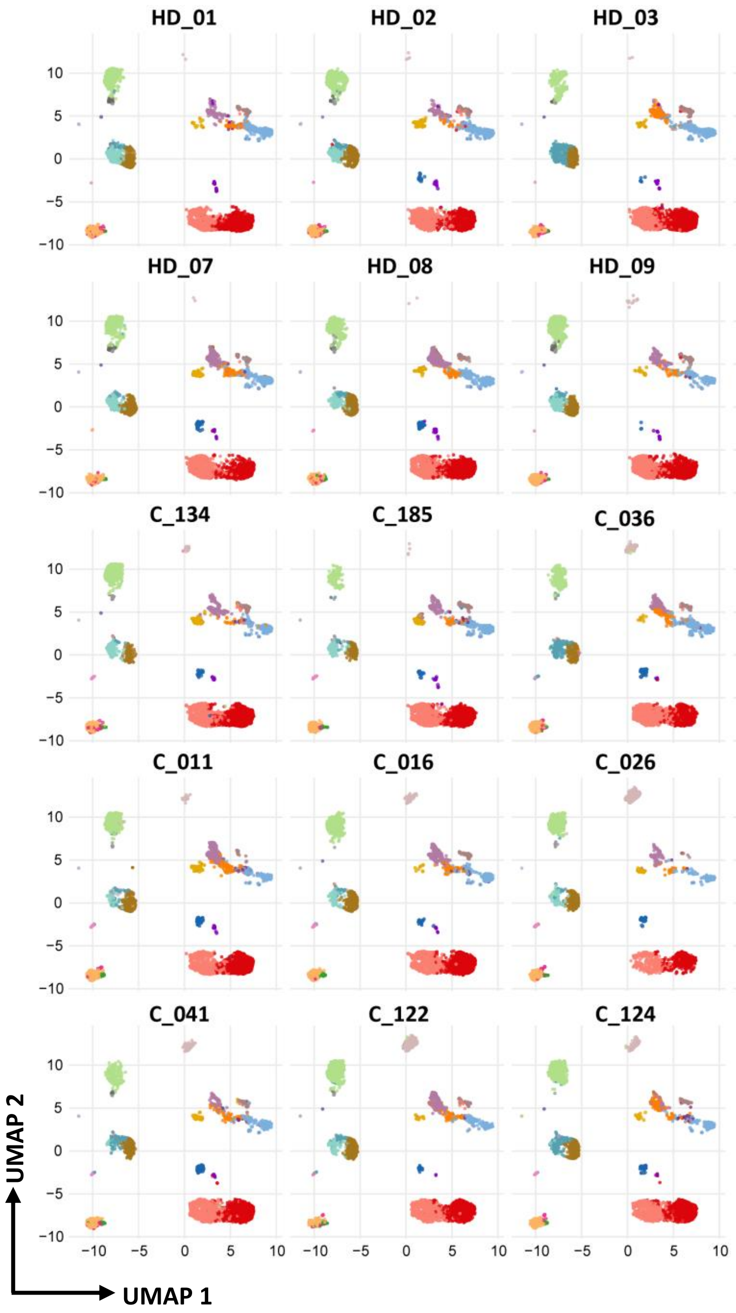

Supplementary Figure 12.
Projection of UMAP graphs stratified by sample embedded with FlowSOM clusters.

a)

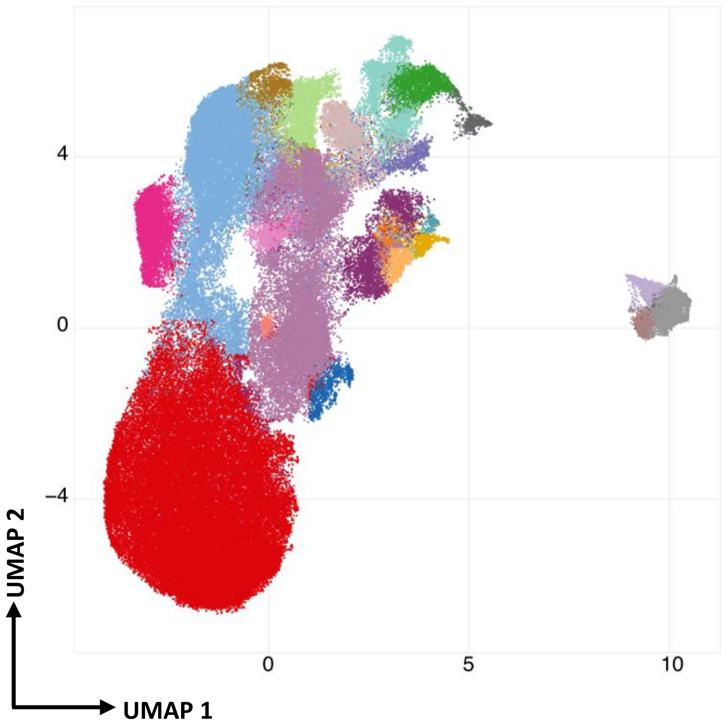

b)

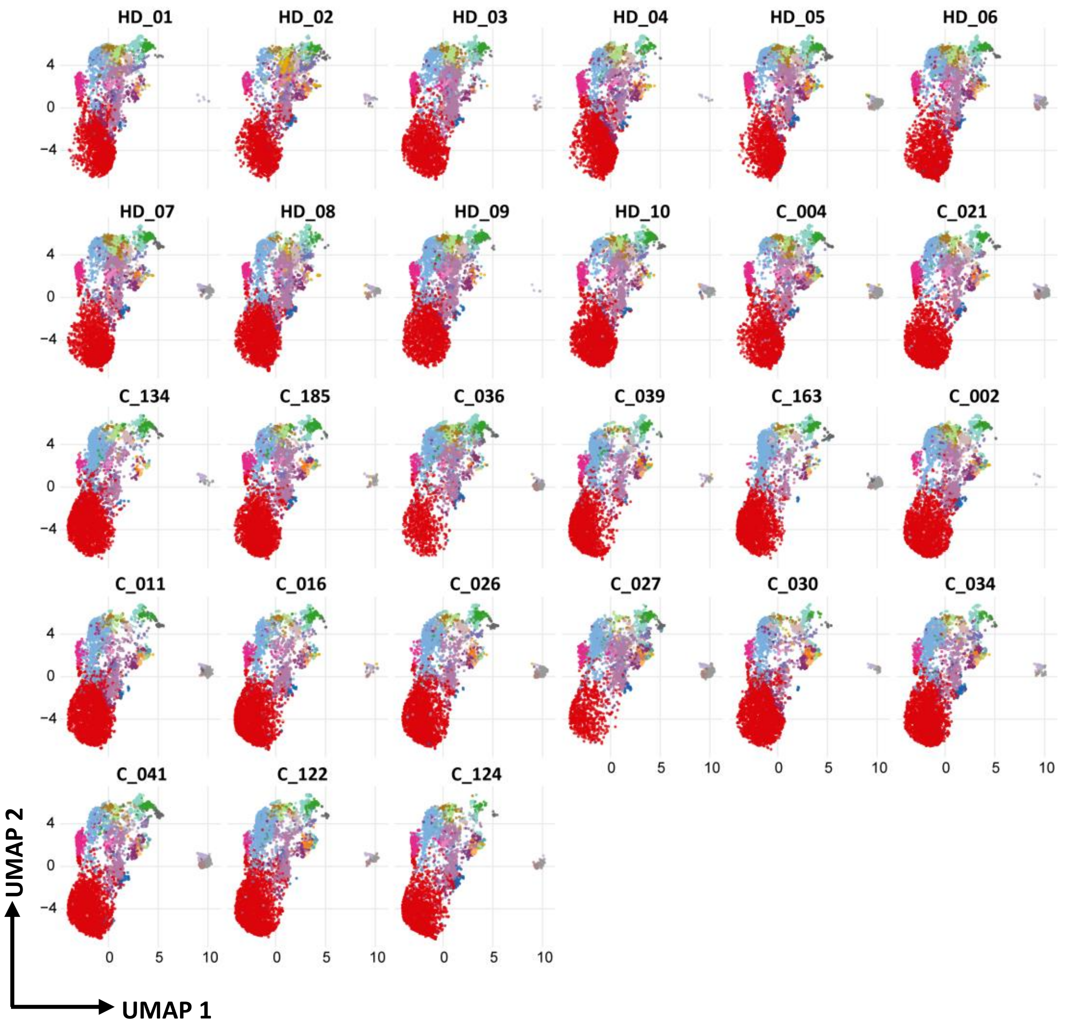

Supplementary Figure 13.
a) UMAP graph of 4,252,208 CD4+ T cells overlaid with FlowSOM clusters; a) Projection of UMAP graph stratified by sample.

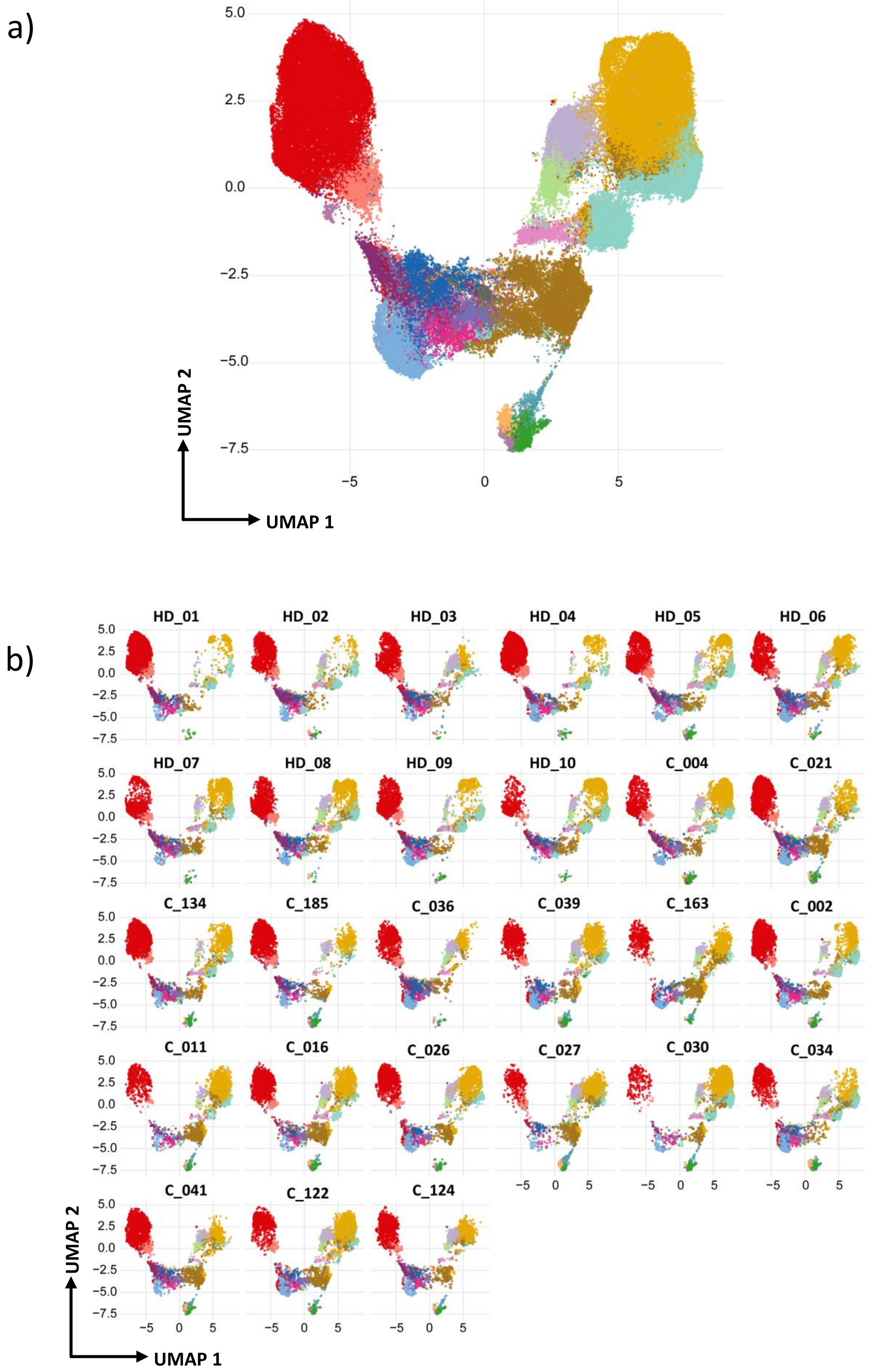

Supplementary Figure 14.
a) UMAP graph of 1,816,526 CD8+ T cells overlaid with FlowSOM clusters; a) Projection of UMAP graph stratified by sample.

a)

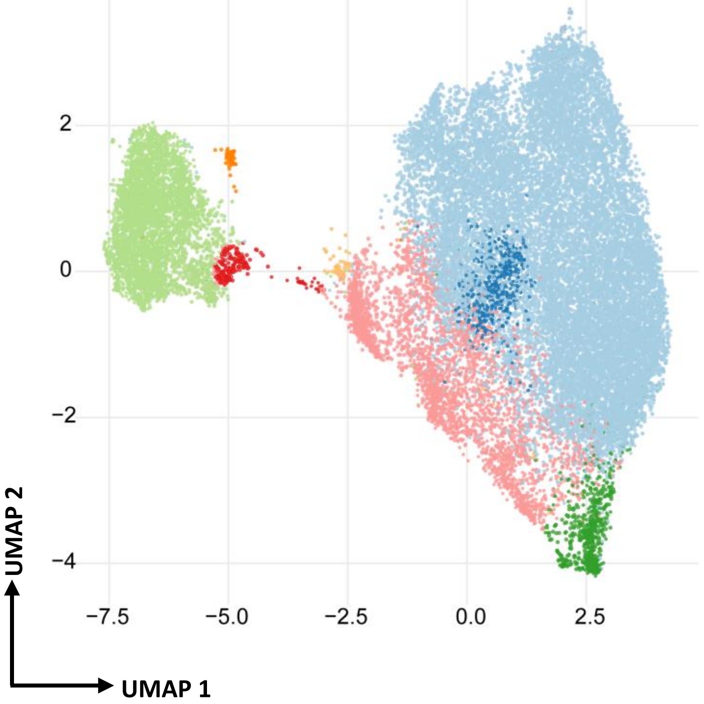

b)

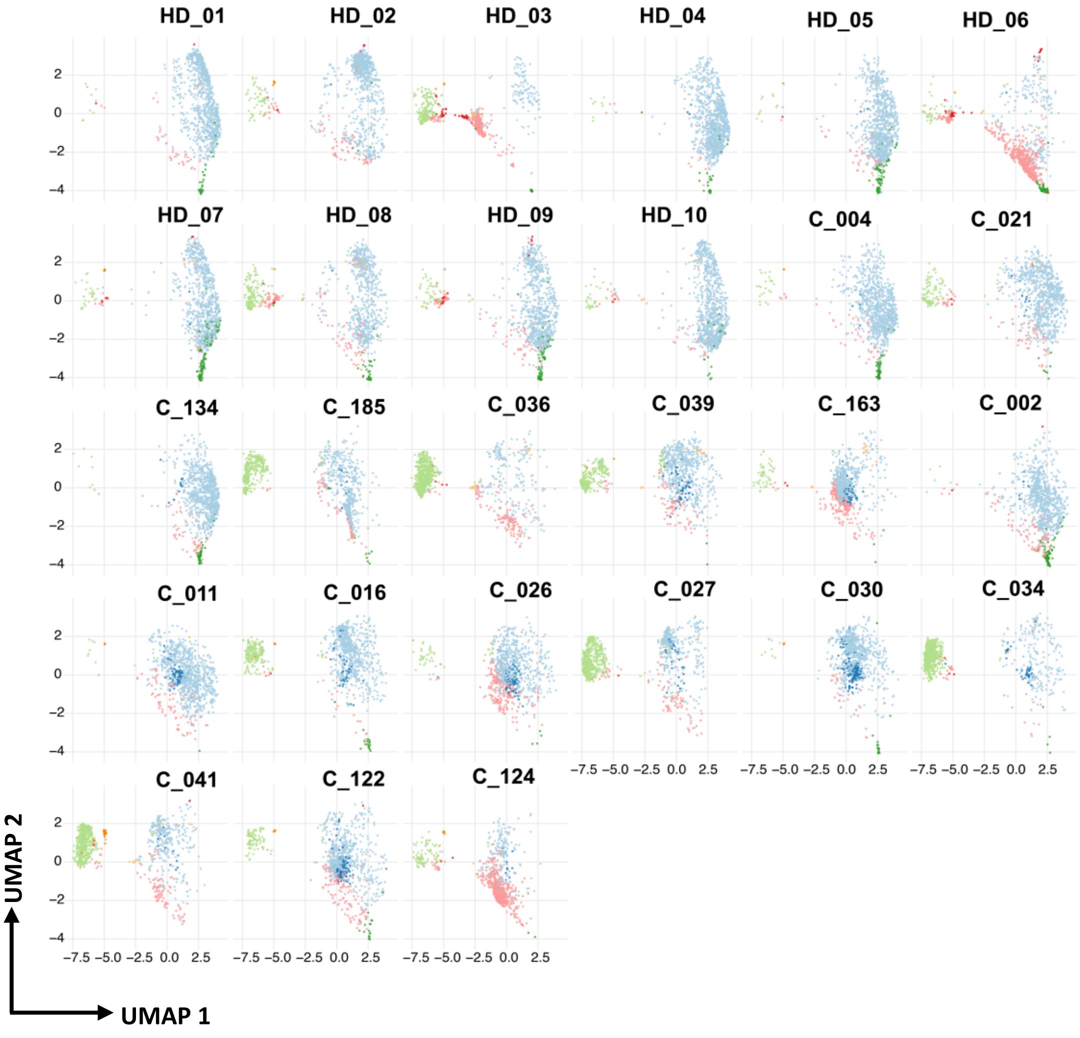

Supplementary Figure 15.
a) UMAP graph of 1,608,200 monocytes overlaid with FlowSOM clusters; a) Projection of UMAP graph stratified by sample.

Supplementary Table 1. List of antibodies used in the CyTOF panel.

Target	Clone	Tag	Note
**CD45**	HI30	89Y	MD-IPA
**CD196 /CCR6**	G034E3	141Pr	MD-IPA
**CD123**	6H6	143Nd	MD-IPA
**CD19**	HIB19	144Nd	MD-IPA
**CD4**	RPA-T4	145Nd	MD-IPA
**CD8a**	RPA-T8	146Nd	MD-IPA
**CD11c**	Bu15	147Sm	MD-IPA
**CD16**	3G8	148Nd	MD-IPA
**CD45RO**	UCHL1	149Sm	MD-IPA
**CD45RA**	HI100	150Nd	MD-IPA
**CD161**	HP-3G10	151Eu	MD-IPA
**CD194/CCR4**	L291H4	152Sm	MD-IPA
**CD25**	BC96	153Eu	MD-IPA
**CD27**	O323	154Sm	MD-IPA
**CD57**	HCD57	155Gd	MD-IPA
**CD183/CXCR3**	G025H7	156Gd	MD-IPA
**CD185/CXCR5**	J252D4	158Gd	MD-IPA
**CD28**	CD28.2	160Gd	MD-IPA
**CD38**	HB-7	161Dy	MD-IPA
**CD56/NCAM**	NCAM16.2	163Dy	MD-IPA
**TCRγδ**	B1	164Dy	MD-IPA
**CD294**	BM16	166Er	MD-IPA
**CD197/CCR7**	G043H7	167Er	MD-IPA
**CD14**	63D3	168Er	MD-IPA
**CD3**	UCHT1	170Er	MD-IPA
**CD20**	2H7	171Yb	MD-IPA
**CD66b**	G10F5	172Yb	MD-IPA
**HLA-DR**	LN3	173Yb	MD-IPA
**IgD**	IA6-2	174Yb	MD-IPA
**CD127**	A019D5	176Yb	MD-IPA
**Cell-ID Intercalator-103Rh**		103Rh	MD-IPA
**CD181/CXCR1**	8F1/CXCR1	142Nd	3142009B
**CD274/PDL1**	29E.2A3	159Tb	3159029B
**CD80/B7.1**	2D10.4	162Dy	3162010B
**CD40**	5C3	165Ho	3165005B
**CD24/PD-1**	EH12.2H7	175Lu	3175008B
**CD11b/Mac-1**	ICRF44	209Bi	3209003B
**CD21**	NA	116Cd	Custom
**IgM**	NA	114Cd	Custom

Supplementary Table 2. List of antibodies used in the flow cytometry panel to measure CFSE dilution.

Target	Dye	Clone	Producer	Catalog Number	Lot Number	Titer(μL)/100μL
**LIVE DEAD**	AQUA	N/A	ThermoFisher	L34966	2268307	1.25
**CFSE**	CellTrace (AF488)	N/A	ThermoFisher	C34554	2208523	1.0
**CD19**	BV605	SJ25C1	BioLegend	363024	B248240	2.0
**CCR7**	PE	G043H7	BioLegend	353204	B311817	3.75
**CD45RA**	PB	HI100	BioLegend	304123	B190975	2.5
**CD4**	APC-H7	RPA-T4	BD	560155	7062573	1.25
**CD8**	APC	SK1	BioLegend	344721	B304310	1.25

Supplementary Table 3. List of antibodies used in the flow cytometry panel to identify cTfh.

Target	Dye	Clone	Producer	Catalog Number	Lot Number	Titer(μL)/100μL
**LIVE DEAD**	AQUA	N/A	ThermoFisher	L34966	2268307	1.25
**CXCR5**	APC	J252D4	BioLegend	356908	B316958	1.25
**PD-1**	BV605	EH12.2H7	BioLegend	329924	B293223	2.5
**CCR7**	FITC	G043H7	BioLegend	353216	B232796	1.25
**CD45RA**	PE-Cy7	HI100	BioLegend	304126	B248293	1.25
**CD3**	PB	UCHT1	Becton Dickinson	558117	6049729	2.5
**CD4**	AF700	RPA-T4	BioLegend	300526	B274110	0.6
**CD8**	APC-Cy7	RPA-T8	BioLegend	301016	B274260	0.6
