# Supplementary material for: Molecular and cellular immune features of aged patients with severe COVID-19 pneumonia
Source: Commun Biol. 2022 Jun 16;5:590. doi: 10.1038/s42003-022-03537-z (PMC9203559; doi:10.1038/s42003-022-03537-z)
Supplement: Supplementary file 3 — Description of Additional Supplementary Files [file 42003_2022_3537_MOESM3_ESM.pdf]

Description of Additional Supplementary Files

File name: Supplementary Data 1
Description: Demographic and clinical characteristics of patients (CUN and COV).

File name: Supplementary Data 2
Description: The source data behind the Figure 3. Differentially expressed genes (DEGs) in CM CD4+ T cells in COV and CUN.
