# Supplementary material for: Molecular and cellular immune features of aged patients with severe COVID-19 pneumonia
Source: Commun Biol. 2022 Jun 16;5:590. doi: 10.1038/s42003-022-03537-z (PMC9203559; doi:10.1038/s42003-022-03537-z)
Supplement: Supplementary file 6 — Reporting Summary [file 42003_2022_3537_MOESM6_ESM.pdf]

nature portfolio

Corresponding author(s): Sara De Biasi, Andrea Cossarizza

Last updated by author(s): May 5, 2022

Reporting Summary

Nature Portfolio wishes to improve the reproducibility of the work that we publish. This form provides structure for consistency and transparency in reporting. For further information on Nature Portfolio policies, see our Editorial Policies and the Editorial Policy Checklist.

Statistics

For all statistical analyses, confirm that the following items are present in the figure legend, table legend, main text, or Methods section.

n/a	Confirmed	
☐	☒	The exact sample size (*n*) for each experimental group/condition, given as a discrete number and unit of measurement
☐	☒	A statement on whether measurements were taken from distinct samples or whether the same sample was measured repeatedly
☐	☒	The statistical test(s) used AND whether they are one- or two-sided<br>*Only common tests should be described solely by name; describe more complex techniques in the Methods section.*
☒	☐	A description of all covariates tested
☐	☒	A description of any assumptions or corrections, such as tests of normality and adjustment for multiple comparisons
☐	☒	A full description of the statistical parameters including central tendency (e.g. means) or other basic estimates (e.g. regression coefficient) AND variation (e.g. standard deviation) or associated estimates of uncertainty (e.g. confidence intervals)
☐	☒	For null hypothesis testing, the test statistic (e.g. *F*, *t*, *r*) with confidence intervals, effect sizes, degrees of freedom and *P* value noted<br>*Give P values as exact values whenever suitable.*
☒	☐	For Bayesian analysis, information on the choice of priors and Markov chain Monte Carlo settings
☒	☐	For hierarchical and complex designs, identification of the appropriate level for tests and full reporting of outcomes
☒	☐	Estimates of effect sizes (e.g. Cohen's *d*, Pearson's *r*), indicating how they were calculated

Our web collection on statistics for biologists contains articles on many of the points above.

Software and code

Policy information about availability of computer code

Data collection	Data in FCS file format were normalized for intra-file and inter-file signal drift using the FCS Processing tab in the CyTOF Software 6.7.<br>PBMC were acquired by using Attune NxT acoustic focusing flow cytometer (ThermoFisher).
Data analysis	Compensated and normalized Flow Cytometry Standard (FCS) 3.0 files were imported into FlowJo software version 10.<br>The main cell population identification was performed through unsupervised clustering using the FlowSOM<br>algorithm (K= 30). 2D visual representation was performed applying Uniform Manifold Approximation and Projection (UMAP). Statistical analysis was performed using generalized linear mixed models (GLMM) applying as FDR cutoff = 0.05.<br>All living CD45+ were exported for further analysis in R using Bioconductor libraries CATALYST (version 1.12.2) 21 and diffcyt (version 1.8.8).<br>Pairwise correlations between variables were calculated and visualized as a correlogram using R function corrplot.<br>Quantitative variables were compared using Mann-Whitney test. Statistical analyses were performed using Prism 8.0

For manuscripts utilizing custom algorithms or software that are central to the research but not yet described in published literature, software must be made available to editors and reviewers. We strongly encourage code deposition in a community repository (e.g. GitHub). See the Nature Portfolio guidelines for submitting code & software for further information.

Data

Policy information about availability of data

All manuscripts must include a data availability statement. This statement should provide the following information, where applicable:

- Accession codes, unique identifiers, or web links for publicly available datasets
- A description of any restrictions on data availability
- For clinical datasets or third party data, please ensure that the statement adheres to our policy

Mass cytometry (CyTOF) data files are available in the flowrepository.org database under accession code FR-FCM-Z48K (https://flowrepository.org/id/FR-FCM-Z48K) except for three files (CTR_1_LG.fcs, CTR_2_RB.fcs, CTR_7_FA.fcs) that are in the folder FR-FCM-Z3GH (https://flowrepository.org/id/FR-FCM-Z3GH). The raw data generated in this study are provided in the Source Data file. Transcriptomic data (RNA-seq) has been deposited in the Gene Expression Omnibus (GEO) public repository under the accession number GSE181005.

Field-specific reporting

Please select the one below that is the best fit for your research. If you are not sure, read the appropriate sections before making your selection.

☒ Life sciences ☐ Behavioural & social sciences ☐ Ecological, evolutionary & environmental sciences

For a reference copy of the document with all sections, see nature.com/documents/nr-reporting-summary-flat.pdf

Life sciences study design

All studies must disclose on these points even when the disclosure is negative.

Sample size	No sample size calculation
Data exclusions	No data were excluded from the analysis.
Replication	Not applicable
Randomization	Not applicable
Blinding	Not applicable

Reporting for specific materials, systems and methods

We require information from authors about some types of materials, experimental systems and methods used in many studies. Here, indicate whether each material, system or method listed is relevant to your study. If you are not sure if a list item applies to your research, read the appropriate section before selecting a response.

Materials & experimental systems

n/a	Involved in the study	
☐	☒	Antibodies
☒	☐	Eukaryotic cell lines
☒	☐	Palaeontology and archaeology
☒	☐	Animals and other organisms
☐	☒	Human research participants
☐	☒	Clinical data
☒	☐	Dual use research of concern

Methods

n/a	Involved in the study	
☒	☐	ChIP-seq
☐	☒	Flow cytometry
☒	☐	MRI-based neuroimaging

Antibodies

Antibodies used	All the antibodies used in the study are reported in Supplementary tables.
Validation	All the antibodies used in the study are commercially available and they have been validated by producers.

Human research participants

Policy information about studies involving human research participants

Population characteristics	Sixty-four COVID-19 patients with severe symptoms were enrolled in the study. Patients were admitted to the Infectious Diseases Clinics or to the Intensive Care Unit (ICU) of the Azienda Ospedaliero Universitaria (University Hospital) in Modena; all patients had a positive SARS-CoV-2 PCR test. Each participant, including healthy donors, provided informed consent according to Helsinki Declaration, and all uses of human material have been approved by the local Ethical Committee (Comitato Etico dell'Area Vasta Emilia Nord, protocol number 177/2020, March 11th, 2020) and by the University Hospital Committee (Direzione Sanitaria dell'Azienda Ospedaliero-Universitaria di Modena, protocol number 7531, March 11th, 2020). Plasma samples and cells from a total of 32 adult healthy donors (HD, mean age of 54.1±15.9) with no prior diagnosis or recent symptoms consistent with SARS-CoV-2 illness and with a negative serology were used for some analyses.
Recruitment	Patients were eligible for inclusion if they were aged 18 years or older, able to provide informed consent and diagnosed with SARS- CoV-2 infection. Confirmed SARS-CoV-2 infection was defined as nasopharyngeal swab reverse transcription–polymerase chain reaction (RT-PCR) test results positive for SARS-CoV-2.
Ethics oversight	This is a case-control, cross sectional, single-centre study, approved by the local Ethical Committee (Comitato Etico dell'Area Vasta Emilia Nord, protocol number 177/2020, March 11th, 2020) and by the University Hospital Committee (Direzione Sanitaria dell'Azienda Ospedaliero-Universitaria di Modena, protocol number 7531, March 11th, 2020).

Note that full information on the approval of the study protocol must also be provided in the manuscript.

Clinical data

Policy information about clinical studies

All manuscripts should comply with the ICMJE guidelines for publication of clinical research and a completed CONSORT checklist must be included with all submissions.

Clinical trial registration	Not applicable
Study protocol	Not applicable
Data collection	Not applicable
Outcomes	Not applicable

Flow Cytometry

Plots

Confirm that:

☒ The axis labels state the marker and fluorochrome used (e.g. CD4-FITC).

☒ The axis scales are clearly visible. Include numbers along axes only for bottom left plot of group (a 'group' is an analysis of identical markers).

☒ All plots are contour plots with outliers or pseudocolor plots.

☒ A numerical value for number of cells or percentage (with statistics) is provided.

Methodology

Sample preparation	Blood samples (up to 20 mL) were obtained after informed consent. In some donors, blood was obtained after diagnosis of SARS-CoV-2 infection. Peripheral blood mononuclear cells (PBMC) were isolated according to standard procedures and stored in liquid nitrogen until use. Plasma was collected and stored at -80°C until use. Measurements were taken from individual patients; in the case of plasma, each measurement was performed in duplicate and only the mean was considered and shown.
Instrument	Attune NxT acoustic flow Cytometer (ThermoFisher), CyTOF Helios (Fluidigm).
Software	FlowJo X, Prism 8.0, Attune NxT software, CyTOF Software 6.7
Cell population abundance	Not applicable
Gating strategy	Al the gating strategy are represented and described in the Supplementary Infomation.

☒ Tick this box to confirm that a figure exemplifying the gating strategy is provided in the Supplementary Information.
